# The mechanisms of colorectal cancer cell mesenchymal-epithelial transition induced by hepatocyte exosome-derived miR-203a-3p

**DOI:** 10.1186/s12885-021-08419-x

**Published:** 2021-06-19

**Authors:** Heyang Xu, Qiusheng Lan, Yongliang Huang, Yang Zhang, Yujie Zeng, Pengwei Su, Ziqiang Chu, Wei Lai, Zhonghua Chu

**Affiliations:** 1 Guangdong Provincial Key Laboratory of Malignant Tumor Epigenetics and Gene Regulation, Department of Gastrointestinal Surgery Sun Yat-sen Memorial Hospital, Sun Yat-sen University, Guangzhou, China; 2 Department of General Surgery, Foshan Maternal and Child Health Hospital, Southern Medical University, Foshan, China; 3 Guangzhou Blood Center, Guangzhou, Guangdong Province China

**Keywords:** miR-203a-3p, Exosome, Mesenchymal-to-epithelial transition, Colorectal cancer

## Abstract

**Background:**

Liver metastasis is the most common cause of death in patients with colorectal cancer (CRC). Phosphatase of regenerating liver-3 induces CRC metastasis by epithelial-to-mesenchymal transition, which promotes CRC cell liver metastasis. Mesenchymal-to-epithelial transition (MET), the opposite of epithelial-to-mesenchymal transition, has been proposed as a mechanism for the establishment of metastatic neoplasms. However, the molecular mechanism of MET remains unclear.

**Methods:**

Using Immunohistochemistry, western blotting, invasion assays, real-time quantitative PCR, chromatin immunoprecipitation, luciferase reporter assays, human miRNA arrays, and xenograft mouse model, we determined the role of hepatocyte exosome-derived miR-203a-3p in CRC MET.

**Results:**

In our study, we found that miR-203a-3p derived from hepatocyte exosomes increased colorectal cancer cells E-cadherin expression, inhibited Src expression, and reduced activity. In this way miR-203a-3p induced the decreased invasion rate of CRC cells.

**Coclusion:**

MiR-203a-3p derived from hepatocyte exosomes plays an important role of CRC cells to colonize in liver.

## Introduction

Liver metastasis is the main cause of death in patients with colorectal cancer (CRC). Once patients are diagnosed with colorectal liver metastases, the 5-year survival rate is 11.7% [1, 2] .Therefore, it is important to successfully identify the signaling pathway leading to CRC cell seeding in the liver; this information may pave the way to find effective therapies for patients with colorectal liver metastases. Studies have revealed that epithelial-to-mesenchymal transition (EMT) occurs in the process of CRC liver metastasis [3]. Further studies indicated that during the process of EMT, epithelial cells lose their epithelial traits and acquire mesenchymal characteristics to gain migratory and invasive properties [4]. At the same time, the morphology of tumor cells changes from epithelial-like to fibroblast-like, which may favor invasion and metastasis and allow the cells to establish secondary tumors at distant sites [5]. It has been demonstrated that cancer cells down-regulate E-cadherin, which is a hallmark of EMT and is associated with tumor cell invasion and metastasis [6]. However, once cancer cells colonize to specific organs, the re-expression of E-cadherin can be observed in the metastasis sites, indicating that cancer cells undergo the phenotypic mesenchymal-to-epithelial transition (MET), which is regulated by the tumor microenvironment.

Phosphatase of regenerating liver-3 (PRL-3) belongs to the protein tyrosine phosphatase family, which plays critical roles in the signal induction leading to the tyrosine phosphorylation of down-stream molecules [7]. PRL-3 has been shown to be overexpressed in the liver metastases of CRC but seldom expressed in the corresponding primary tumors or normal colorectal epithelium, indicating that it participated in the progression and metastasis of tumor cells [8]. Recently, considerable evidence has suggested that EMT is the mechanism by which PRL-3 promotes CRC metastasis. For example, PRL-3 regulated the phosphoinositide 3-kinase (PI3k)/AKT pathway to modulate E-cadherin expression [9]. More obviously, PRL-3 can regulate cadherin directly to enhance the invasion ability of CRC [10]. The expression of PRL-3 has been found to be positively correlated with tumorigenesis and metastasis in various tumors, including CRC, gastric cancer and ovarian cancer [11–13]. Our previous study also revealed that PRL-3 could activate the NF-κB pathway to induce KCNN4 expression, leading to the inhibition of E-cadherin expression and the promotion of CRC liver metastasis [14]. Although various signaling pathways have been implicated in PRL-3-induced EMT and it is well known that cancer cells need to undergo MET before colonizing the liver, whether PRL-3 regulates the progression of MET remains unknown. A recent study has revealed that PRL-3 could induce the activation of the epidermal growth factor receptor (EGF)/EGFR (epidermal growth factor receptor) signaling pathway; this finding indicates that PRL-3 may be involved in the progression of MET [15].

EGFR is a receptor tyrosine kinase that can initiate pleiotropic intracellular signaling leading to defects associated with pathologies such as cancer [16]. Once EGFR signaling is activated, the downstream molecules, such as mitogen-activated protein kinases, PI3K-AKT, and Jak-Stat, are also activated [17]. The EGFR pathway has emerged as a key anticancer target for blocking the invasion and proliferation of tumor cells. Furthermore, it has been shown that effective targeted therapies against EGFR depend on the KRAS and BRAF status in patients with metastatic CRC and lung cancer, indicating that mutations could influence the activation of the EGFR pathway [18]. The EGF/EGFR signaling pathway was also found to induce EMT in several tumor types, including CRC, through the regulation of E-cadherin expression [19]. Interestingly, when cancer cells were co-cultured with hepatocytes, the activation of the EGFR signaling pathway was inhibited. At the same time, the expression of E-cadherin was induced, indicating that the EGF/EGFR pathway can regulate E-cadherin expression [20]. However, the specific mechanism is unclear.

Exosomes are small vesicles secreted by cell endosomes that range between 40 and 100 nm in diameter [21]. Studies have found that exosomes can regulate target cells directly through receptor combination or by transferring various bioactive molecules, such as proteins, mRNAs and circRNAs, into the target cells [22]. MicroRNAs (miRNAs) are non-coding single-stranded RNAs that are encoded by endogenous genes. miRNAs has been shown to play an important regulatory role in gene expression by binding to the 3′-UTRs (untranslated regions) of their target mRNAs [23]. It has been demonstrated for the first time that the dysregulation of miRNAs plays a very important role in the development of tumors [24]. Regarding tumor metastasis, miRNAs, such as miR-155, miRNA-200, and miR-205, can promote/inhibit the metastasis of malignant tumors by regulating EMT through RhoA or by regulating the expression of the E-cadherin transcription receptors ZEB1 and ZEB2 [25].

In this study, we aimed to explore the mechanisms of CRC cell MET, which facilitates cancer cell colonization to the liver.

## Materials and methods

### Cells and cell culture/co-culture conditions

Stable cells (vector-transfected and PRL-3-expressing cells) were established using LoVo cells (Guangzhou Cellcook Biotech Co. Ltd) as described previously [26]. G418 cells were purchased from Sigma (St Louis, MO, USA). Cells were maintained in RPMI 1640 (HyClone, Logan, UT, USA) with 10% fetal bovine serum (FBS; Gibco, Carlsbad, CA, USA) at 37 °C in a humidified incubator with 5% CO_2_. The LO2 cell line (Guangzhou Cellcook Biotech Co. Ltd) was maintained in DMEM (HyClone, Logan, UT) containing 10% FBS. Co-culture analysis was performed in 6-well plates with transwell chambers (0.4-μm pore size, Corning, Canton, NY, USA). Cancer cells (1 × 10^5^) were plated in the lower chamber of each well in 2000 μL of complete medium with 10% FBS and allowed to attach overnight. The next day, LO2 cells were plated at 5 × 10^4^ cells per well in 1500 μL of complete medium in the upper chambers. The medium was replenished every 2 days. The PKC inhibitor GF109203X was obtained from Sigma. Recombinant human EGF protein was obtained from PeproTech (Rocky Hill, NJ, USA).

### CRC samples

CRC samples were obtained from patients who were diagnosed with CRC and then underwent elective surgery at the Department of Gastroenteropancreatic Surgery, Sun Yat-sen Memorial Hospital, Sun Yat-sen University, between July 2006 and June 2010. The protocol was approved by the Ethics Committee of Sun Yat-Sen Memorial Hospital, China (Approve No.179). Seventy-five primary CRC samples (stage I, *n* = 12; stage II, *n* = 15; stage III, *n* = 25 and stage IV, *n* = 23) and 23 corresponding colorectal liver metastasis samples from stage IV patients were obtained from surgically resected specimens for immunohistochemistry.

### Immunohistochemistry

Paraffin-embedded patient samples were obtained from Sun Yat-Sen Memorial Hospital. Colorectal tumor specimens were fixed in formalin and embedded in paraffin. After that, the tissue sections were deparaffinized in xylene for 10 min and then subjected to antigen retrieval by boiling in 0.01 M citric buffer (pH 6.0). Endogenous peroxidase activity in the samples was blocked with 3% hydrogen peroxide in PBS and 0.05% Tween 20 for 30 min. The samples were then washed with PBS and blocked for 30 min with 20% normal goat serum at room temperature, followed by incubation with primary antibodies against PRL-3, p50, p65 (Abcam, Cambridge, MA, USA), E-cad (Santa Cruz, California, CA, USA), p-EGFR and VEGF-A (Cell Signaling Technology, Cambridge, MA, USA) at a dilution of 1:100 in a humidified chamber overnight at 4 °C. The next day, the sections were washed with PBS, incubated with peroxidase-conjugated secondary antibodies for 2 h at room temperature and then rinsed with PBS. Finally, the DAB Plus substrate staining system (Abcam, Cambridge, MA, USA) was used to stain the samples according to the manufacturer’s instructions. All tissue sections were counterstained with hematoxylin and mounted with aqueous mounting media. The tissue sections were scored quantitatively according to the percentage of positive cells and staining intensity. The intensity of staining was scored from 0 to 3 (I0, I1–3): 0 (no staining), 1 (weak staining = light yellow), 2 (moderate staining = yellow brown) and 3 (strong staining = brown). The proportion of the tumor stained at a particular intensity was recorded in 5% increments using a range of 0–100 (P0, P1–3). The final H score (range 0–300) was calculated for each intensity and proportion of the area stained (H score = I1 × P1 + I2 × P2 + I3 × P3).

### Western blot analysis

Cells were washed three times with cold PBS and lysed on ice with RIPA buffer containing 1% PMSF and a proteinase inhibitor cocktail. Sample protein concentrations were determined by Bradford assays [26]. Forty-microgram samples for each lane were boiled for 5 min in sample buffer. The denatured proteins were then separated by 10% or 12% sodium dodecyl sulfate-polyacrylamide gel electrophoresis and transferred onto polyvinylidene fluoride membranes. Nonspecific reactivity was blocked using 5% bovine serum albumin in a TBST buffer. The membranes were then incubated with the relevant primary antibodies overnight at 4 °C. Following incubating with a horseradish peroxidase-conjugated secondary antibody at a 1:5000 dilution for 2 h at room temperature, the labeled proteins were visualized by chemiluminescence (American Bioscience, Piscataway, NJ, USA) western blotting detection reagents. Anti-GAPDH (mice, Abcam, Cambridge, MA, USA) was used to ensure equal amounts of protein were present for each sample. The protein amounts were estimated through densitometry as the ratio of the detected protein/GAPDH. Anti-vimentin, anti-EGFR, anti-Snail, anti-p-AKT, anti-AKT, anti- activated extracellular signal-regulated kinase (p-Erk1/2), anti-Erk1/2, anti-p-PKC, anti-PKC, anti-p-GSK-3β and anti-GSK-3β antibodies which were obtained from Abcam (mice, Cambridge, MA, USA). Incubations were substrate with ChemiDoc XRS+ imaging system (Bio-Rad Laboratories, Hercules, CA, USA) were used to detect immunoreactive bands.

### Isolation of exosomes from medium and serum

Exosomes were isolated from cell culture medium by differential centrifugation. After removing cells and other debris by centrifugation at 300×g and 3000×g, the supernatant was centrifuged at 10,000×g for 30 min to remove shedding vesicles and other larger-sized vesicles. Finally, the supernatant was centrifuged at 110,000×g for 70 min (all steps were performed at 4 °C); exosomes were collected from the pellet and re-suspended in PBS. Sr-exosomes were isolated by using an exosome isolation kit (Thermo, Waltham, MA, USA).

### Luciferase assay

The reporter plasmid containing the predicted miR-203a-3p targeting regions was designed by Genescript (Nanjing, China). Part of the wild-type and mutated 3′-UTR of Src was cloned immediately downstream of the firefly luciferase reporter. A total of 2 mg of the β-galactosidase expression vector (Ambion) was used as a transfection control. For the subsequent luciferase reporter assays, 2 mg of the firefly luciferase reporter plasmid, 2 mg of the β-galactosidase vector and equal doses (200 pmol) of the mimics, inhibitors or scrambled negative control RNA were transfected into the prepared cells. At 24 h after transfection, the cells were analyzed by using a dual luciferase assay kit (Promega) according to the manufacturer’s instructions. Each sample was prepared in triplicate, and the entire experiment was repeated three times.

### Invasion assays

Transwell chambers (BD Biosciences, Franklin Lakes, NJ, USA) were used to measure the ability of cells to invade in cell invasion assays. A total of 1 × 10^5^ cells in 0.2 mL of serum-free RPMI 1640 medium were added to the upper chambers (with Matrigel [Collaborative Biomedical Products, Bedford, MA, USA]). The lower chambers contained 0.8 ml of medium with 10% FBS. After incubation at 37 °C and 5% CO_2_ for 24 h, the cells that invaded to the lower chamber were fixed in 4% paraformaldehyde and stained with 0.1% crystal violet in methanol. The cell counts are expressed as the mean number of cells per field of view. Three independent experiments were performed, and the data are presented as the mean standard deviation.

### Immunofluorescence analysis

Cells were grown to 50–70% confluence in glass-bottom dishes (Nest, Wuxi, China), washed three times with PBST, and fixed in 4% PFA. For immunofluorescence staining, the cells were incubated with primary antibodies against E-cadherin, Snail, or PRL-3 (mice, Abcam, Cambridge, MA, USA) at 4 °C overnight. After thorough washing, staining with Alexa-Fluor-488 conjugated goat anti-mouse and Alexa-Fluor-555 conjugated donkey anti-rabbit secondary antibodies was carried out at room temperature for 60 min, followed by DAPI nuclear counterstaining for 10 min. Finally, the cells were washed three times with PBST and photographed under a laser confocal microscope (Zeiss LSM710, Oberkochen, Germany). The data were processed with Adobe Photoshop 7.0 software (Newton, MA, USA).

### RNA isolation and real-time quantitative polymerase chain reaction (PCR)

Total RNA was extracted using TRIzol reagent (Bio-Rad Laboratories, Hercules, CA, USA), and cDNA was synthesized with Prime Script RT (Bio-Rad Laboratories, Hercules, CA, USA) from 500 ng of RNA according to the manufacturer’s protocol. Quantitative real-time PCR for VEGF (GAPDH was used an internal control) was performed using a Light Cycler 480 (Roche, Basel, Switzerland) and SYBR assay (Takara, Dalian, China). Band intensities were analyzed quantitatively using ImageJ software (NIH, Bethesda, USA).

### Human miRNA arrays

For the secreted miRNA analysis, total RNA isolation was performed with the miRNeasy kit (Qiagen, Valencia, CA, USA) according to the manufacturer’s protocol. The concentration and purity of the isolated RNA were determined using a spectrophotometer, and the integrity of the RNA was verified using an Agilent Eukaryote Total RNA Nano Series II chip on an Agilent 2100 BioAnalyzer. miRNA expression profiling of the samples was performed using the Affymetrix GeneChip miRNA 4.0 array platform (Affymetrix, Santa Clara, CA, USA) at the Johns Hopkins Deep Sequencing and Microarray Core (http://www.microarray.jhmi.edu/) according to the manufacturer’s protocol. This array version covers all mature miRNA sequences.

#### Animals

Athymic nude mice were purchased from the Guangdong Provincial Medical Laboratory Animal Center, animal experiments were followed the NIH guidelines (NIH Pub. No. 85–23, revised 1996), and were approved by Animal Ethical and Welfare Committee of Sun Yat-sen University, and were performed according to the arrived guidelines 2.0. Mice were sacrificed by cervical dislocation in anaesthetized condition.

### Xenograft mouse model

Athymic nude mice (BALB/c nu/nu, 6-week-old females) were used for liver metastasis assays via intrasplenic injection. A total of 200 μL of LoVo-P (PRL-3 stably transfected into LoVo cells) or LoVo-C (Control vector stably transfected into LoVo cells) cells at a concentration of 1 × 10^7^ cells/mL was injected into the spleens of nude mice (*n* = 6 per group). The mice were housed in pathogen-free environments; they were checked, and data were recorded every 3 days. All animals were sacrificed on day 36, which were euthanized by an intraperitoneal injection of excessive pentobarbital. Livers and spleens were resected and photographed. For immunohistochemical staining, the livers were fixed in 4% paraformaldehyde and stained as described above.

### Statistical analysis

Statistical analyses were performed using 18.0 SPSS software (SPSS, Chicago, IL, USA). All data are presented as the mean ± SD. Student’s *t*-test was used to compare two independent groups of data. One-way analysis of variance (ANOVA) was used to analyze the significance among groups. Statistical tests for data analysis also included Fisher’s exact and chi-square tests. Bivariate correlations between study variables were calculated by Spearman’s rank correlation coefficients. *P* values < 0.05 were considered statistically significant.

## Results

### Correlation between E-cadherin、vimentin and PRL-3 in primary site of CRC and liver metastases

**Fig. 1:**
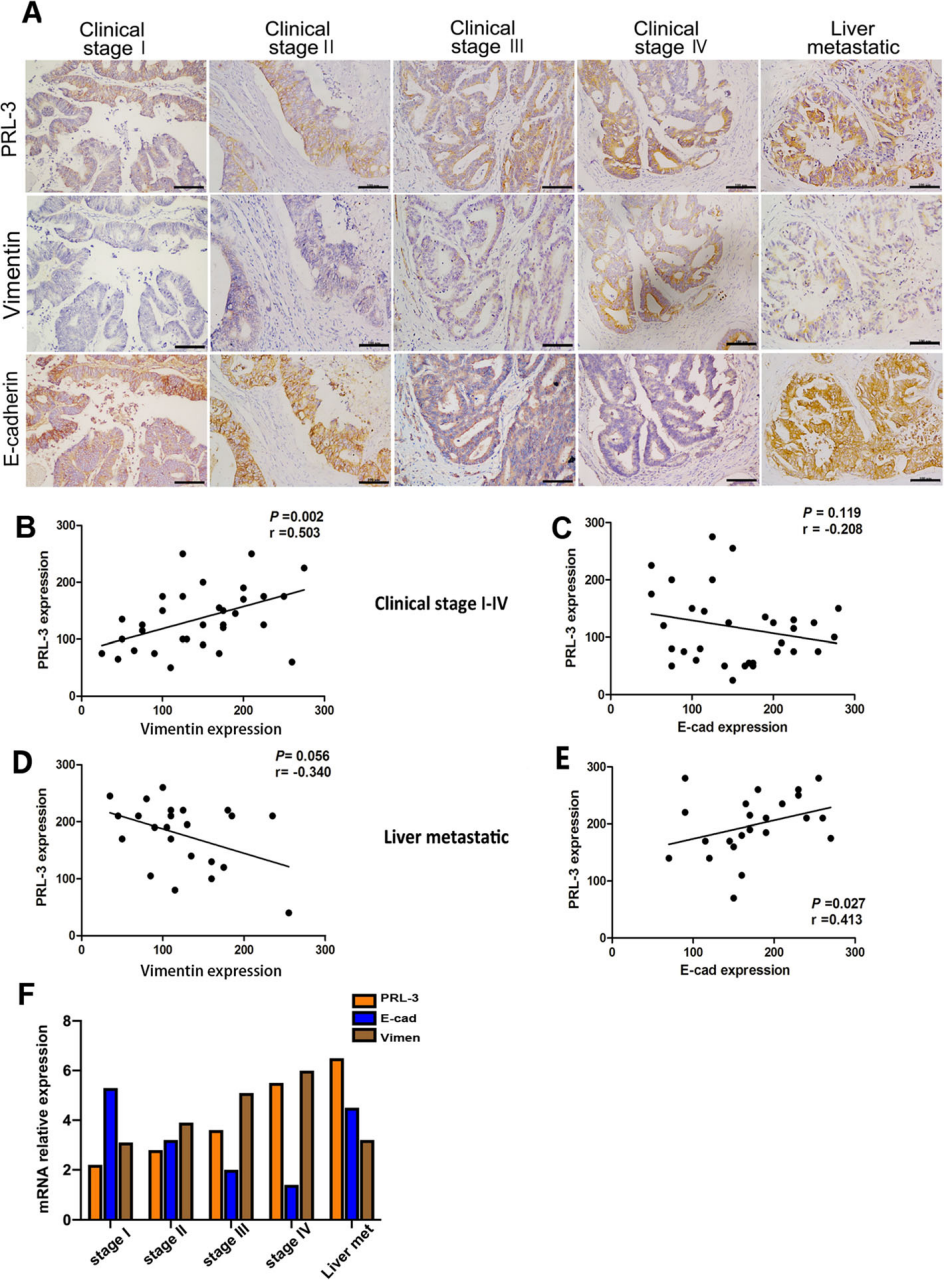
Clinically relevant expression of PRL-3 and E-cadherin in CRC tissues and liver metastases. Percentages of specimens showing low or high PRL-3 expression relative to the level of E-cadherin (E-cad) in CRC tissues (clinical stages I–IV) and in liver metastasis tissues (A). IHC staining shows the immunoreactivity of PRL-3 and E-cad in human CRC (clinical stages I–IV) and liver metastasis tissues. Original magnification, 200×. General correlation between relative PRL-3 and E-cad expression levels in CRC tissues (clinical stages I–IV) and in liver metastases in 23 samples (B-C)

To examine the relationship between the E-cadherin/Vimentin and PRL-3 expression in colorectal tumor and liver metastasis specimens, we performed IHC analyses of 75 human colorectal tumors. The tissue staining was calculated by the percentage of stained cells and the staining intensity. In the primary tumor samples, the level of E-cadherin expression was negatively associated with PRL-3 expression, and Vimentin has a positive correlation with PRL-3. In the colorectal liver metastasis tissues, a statistical analysis indicated an inverse correlation between the levels of E-cadherin/Vimentin and PRL-3 expression (Fig. 1A). However, the re-expression of E-cadherin was detected once CRC cells were colonized to the liver, and Vimentin was reduced. Besides, mRNA expression confirmed same E-cad/Vimentin expression in different CRC stage samples and liver metastasis specimens (Fig. 1B-F). These results suggest that CRC cells undergo a change from EMT to MET in the progression of liver metastases.

### Co-culture with hepatocytes results in the re-expression of E-cadherin in CRC cells

**Fig. 2:**
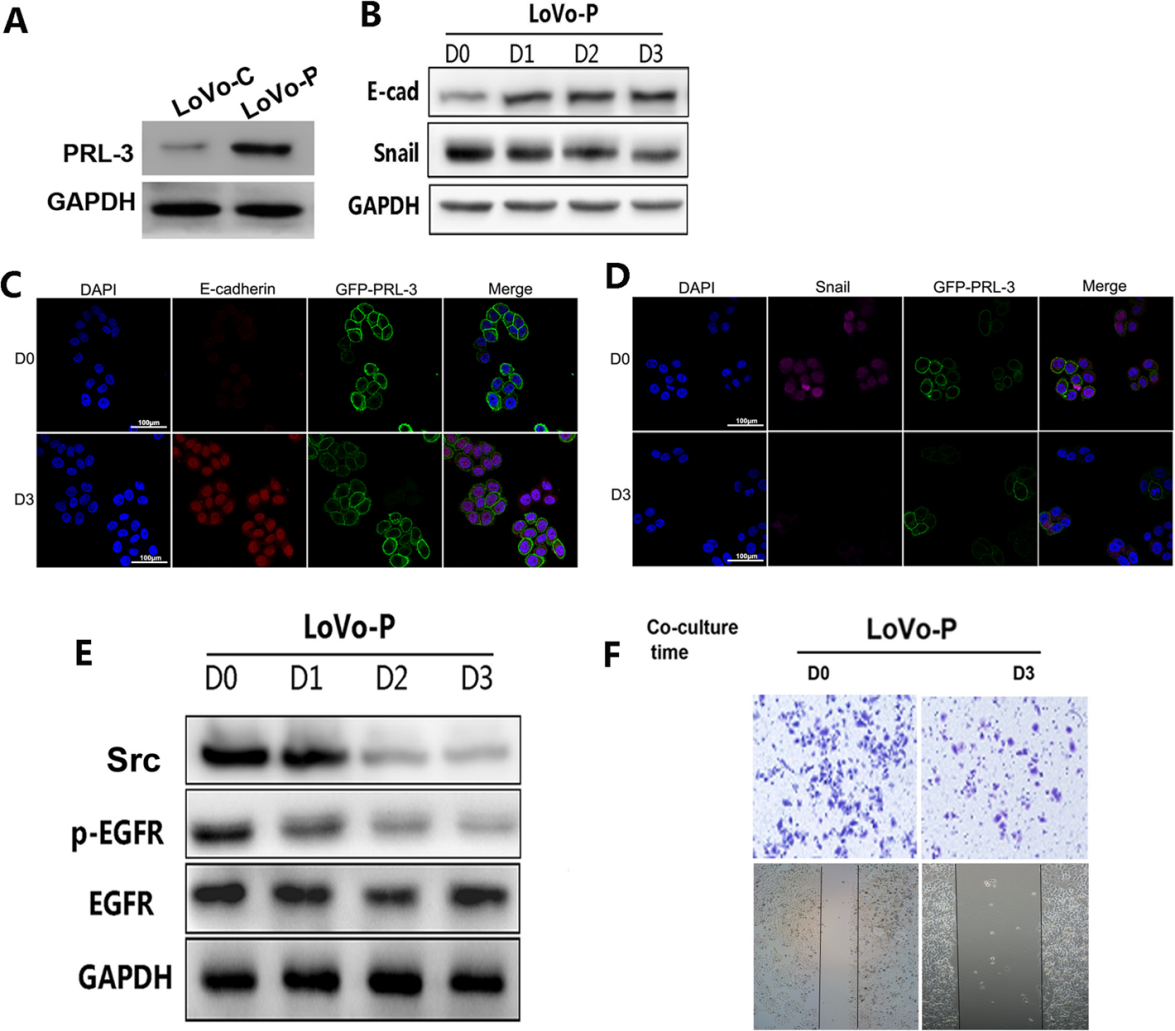
Co-culture with hepatocytes resulted in the re-expression of E-cadherin in CRC cells. (A) PRL-3 expression in stably transfected cell lines (LoVo-P and LoVo-C) was analyzed by western blotting. GAPDH served as the loading control. (B) LoVo-P cells were co-cultured with human hepatic LO2 cells for 0–3 days. The protein expression of E-cad and Snail were analyzed by western blotting. GAPDH served as the loading control. **P < 0.01. The immunoreactivity of E-cad (C) and Snail (D) detected by immunofluorescence staining. The nuclei were visualized with DAPI staining (blue). E-cadherin and Snail were immunostained with red, and PRL-3 was immunostained with green, and merged with blue. Original magnification, 400×. (E) Src and p-EGFR protein expression in LoVo-P cells co-cultured with LO2 cells was evaluated by western blot. (F) Images of invaded cells. Cells were co-cultured with human hepatic LO2 cells for 0/3 days and then harvested to perform invasion assays. The data represent the average of three independent experiments. **P < 0.01

LoVo cells were used due to their low expression of endogenous PRL-3, and these cells were transfected with the PAcGFP-PRL-3/Control vectors (LoVo-P and LoVo-C). The expression of ectopic PRL-3 was verified by both western blot and real-time quantitative RT-PCR assays (Fig. 2A). The expression of E-cadherin was lower in LoVo-P cells as we have previously demonstrated [27]. E-cadherin expression is a typical epithelial cell marker. To determine the role of hepatocytes in the progression of E-cadherin re-expression, we co-cultured LoVo-P cells with LO2 cells. Western blot analyses demonstrated that after co-culture with hepatocytes, the expression of E-cadherin was increased in a time-dependent manner. In contrast, the expression of snail, a prototypical mesenchymal marker, was significantly decreased in a time-dependent manner (Fig. 2B). Immunofluorescence analyses were also carried out to further confirm the re-expression of E-cadherin and the down-regulation of snail (Fig. 2C, D). Since Src plays an important role in regulating EMT through EGFR activation [28], we next examined Src expression and EGFR activation in LoVo-P cells co-cultured with/without LO2 cells. The results showed that Src and EGFR activation were inhibited when LoVo-P cells were co-cultured with LO2 cells (Fig. 2E). As invasion and migration are important consequences of MET, we investigated the impact of LoVo-P cells co-cultured with hepatocytes on invasion with transwell invasion and scratch assays. The results indicated that the invasion and migration rates of LoVo-P cells co-cultured with LO2 cells decreased compared with those of LoVo-P cells cultured alone (Fig. 2F). These results demonstrated that hepatocytes induced a change in co-cultured LoVo-P cells to the epithelial phenotype.

### MiR-203a-3p of hepatocyte-derived exosomes cause the re-expression of E-cadherin through blocking Src expression

**Fig. 3:**
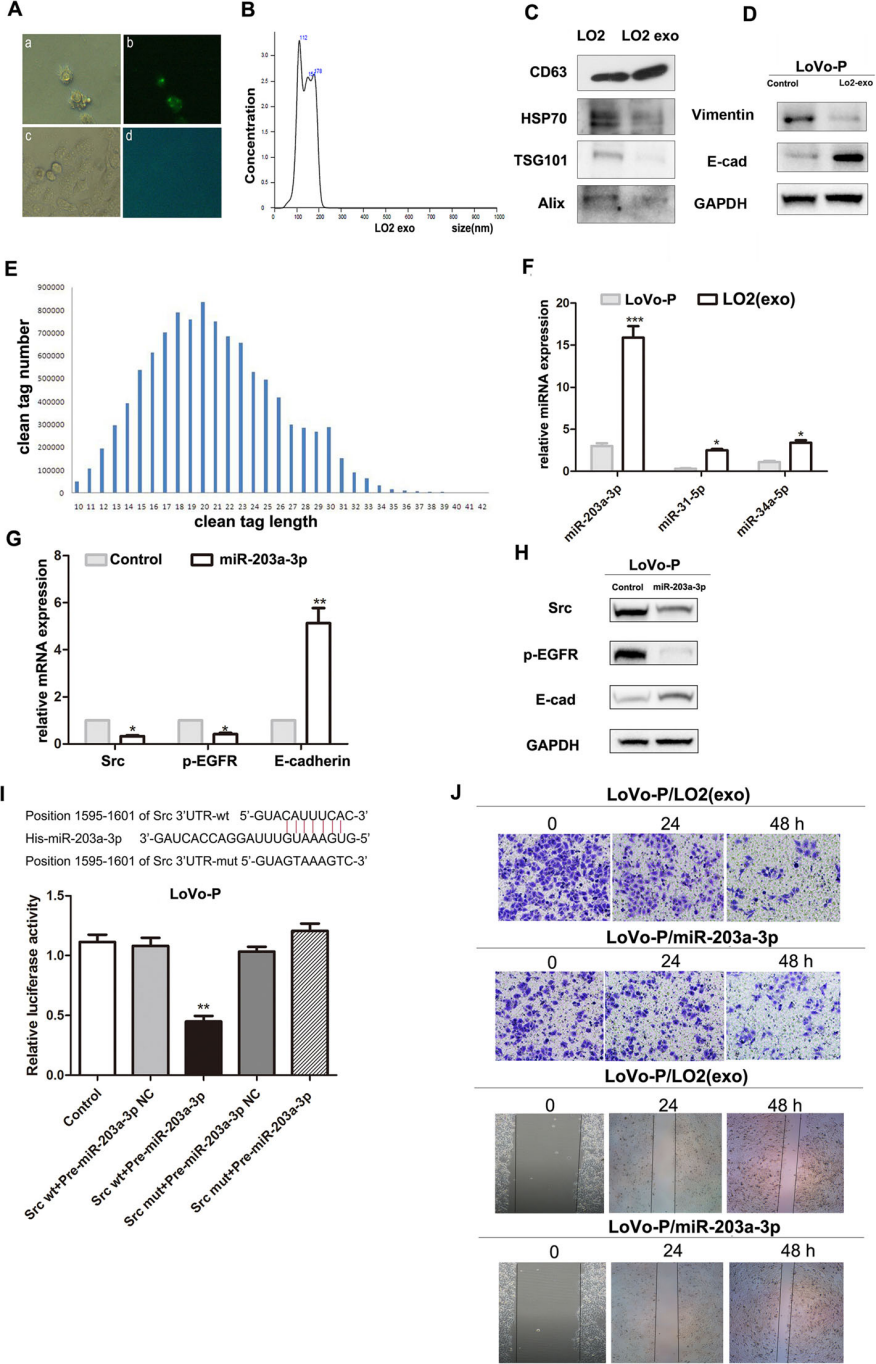
MiR-203a-3p of hepatocyte-derived exosomes caused the re-expression of E-cadherin through blocking Src expression. (A) Exosomes derived from LO2 cells were observed by electron microscopy: a. green labelled exosome from LO2 co-cultured with LoVo-P. b. Green labelled exosome co-cultured with LoVo-P under fluorescence microscope. c. Unlabelled exosome from LO2 co-cultured with LoVo-P. d. Unlabelled exosome from LO2 co-cultured with LoVo-P under fluorescence microscope. (B) Wavelengths of exosomes, size distribution of vesicles identified in the 110,000 g medium pellets of 25 vesicles. (C) CD63, HSP70 and TSG101 which are marker proteins in exosomes were examined by western blot. (D) Expression of E-cad and Vimentin in LoVo-P cells co-cultured with LO2 exosoms. (E-F) RNA was extracted from the LO2 cells 110,000 g medium pellet, a human miRNA array was used to screen miRNA expression in LO2-derived exosomes, the front 1000 kurtosis expression of microRNA were used(E), and the filtered microRNA results were confirmed by qPCR (F). (G-H) An exogenous miR-203a-3p mimic (50 nM) was transfected into LoVo-P cells, qPCR (G) and western blot (H) assays were used to examine the expression of Src, p-EGFR and E-cadherin. (I) Predicted binding sites of miR-203a-3p within the 3′-UTR of Src mRNA. (J) Transwell and scratch assays for number of invaded LoVo-P cells co-cultured with LO2 cells exosomes or transfected with the exogenous miR-203a-3p mimic. The data represent the average of three independent experiments. **P < 0.01 compared with control

Exosomes play an important role in cell to cell communication and interaction. To determine whether hepatocyte-derived exosomes contribute to the change to MET, we first analyzed the structure of LO2-derived exosomes by electron microscopy; the results revealed a typical exosome structure and size of approximately 100 nm (Fig. 3A, B). Exosome protein markers were examined by western blot (Fig. 3C). To define whether LO2- derived exosomes contributed to MET of LoVo-P cells, we first co-cultured exosomes with LoVo-P cells, results showed LoVo-P cells E-cad was elevated and Vimentin was reduced after cocultured with LO2exosomes (Fig. 3D). To determine the key factors of exosomes that inhibit Src expression, we used a human miRNA array and found that miR-203a-3p was increased and could potentially block Src expression (Fig. 3E, F). Then, we transfected an exogenous miR-203a-3p mimic (50 nM) into LoVo-P cells; we found that Src expression and EGFR activation were inhibited, and the expression of E-cadherin was increased (Fig. 3G, H). To provide direct evidence of the interaction between miR-203a-3p and Src, we used a luciferase reporter plasmid containing either the wild-type or mutant 3′-UTR of Src mRNA; the results showed that luciferase activity was reduced markedly in the cells transfected with the exogenous miR-203a-3p mimic, and the inhibitory activity of miR-203a-3p was lost when the binding sites were lost (Fig. 3I). Moreover, invasion and migration were also decreased when the exogenous miR-203a-3p mimic was transfected into LoVo-P cells (Fig. 3J). These results demonstrate that miR-203a-3p increases E-cadherin levels and promotes LoVo-P cell MET through inhibiting Src expression.

### MiR-203a-3p inhibited the Src/PKC/GSK-3β-mediated re-expression of E-cadherin

**Fig. 4:**
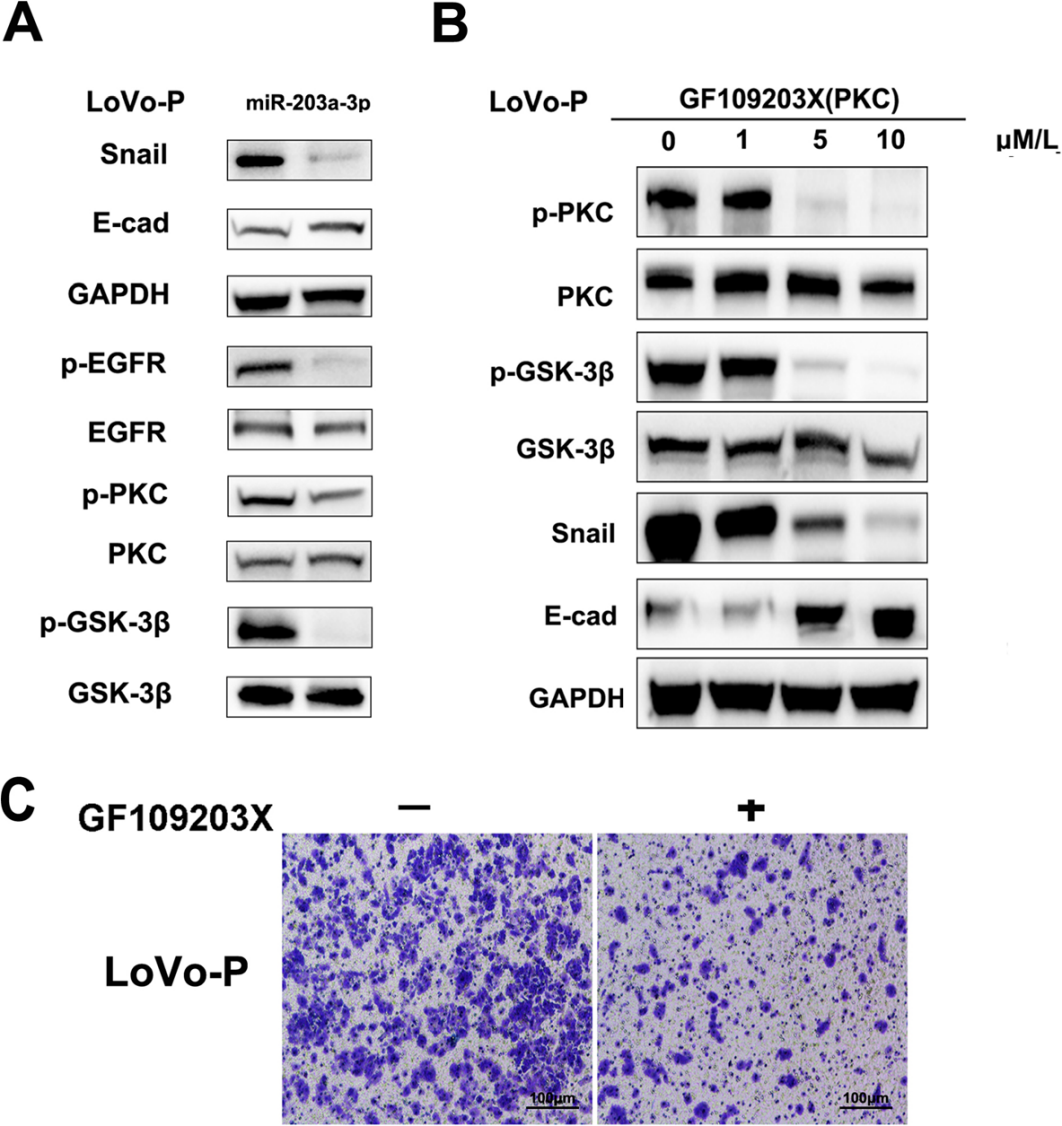
MiR-203a-3p inhibited the Src/PKC/GSK-3β-mediated re-expression of E-cadherin. (A) Expression of E-cadherin, Snail, p-EGFR, p-PKC and p-GSK-3β in LoVo-P cells transfected with the exogenous miR-203a-3p mimic. (B) Expression of E-cadherin, Snail, p-EGFR, p-PKC and p-GSK-3β in LoVo-P cells pretreated with GF109203X. (C) Invaded LoVo-P cells after pretreatment with PKC inhibitor GF109203X and labelled with purple crystal. The data represent the average of three independent experiments. **P < 0.01

To explore the molecular mechanisms responsible for miR-203a-3p-induced MET in CRC cells, we detected the expression of Snail, which has been proven to repress the expression of E-cadherin [28]. The results indicated that the expression of Snail was gradually decreased when LoVo-P cells were transfected with the exogenous miR-203a-3p mimic, and the expression of E-cadherin was increased (Fig. 4A). Because GSK-3β induces the phosphorylation of snail to cause its degradation and because GSK-3β phosphorylation induces GSK-3β inhibition, we further detected the expression of phosphorylated GSK-3β. The results showed that the expression of phosphorylated GSK-3β was decreased; this regulation was governed by PKC activity, which is a downstream molecule of the EGFR signaling pathway. Consistently, PKC phosphorylation was decreased in LoVo-P cells transfected with the exogenous miR-203a-3p mimic (Fig. 4A).

To further confirm whether PKC activity was responsible for the Snail and E-cadherin regulation leading to MET, the PKC kinase inhibitor GF109203X was used. While PKC activation via phosphorylation was significantly reduced by PKC inhibitor treatment at the indicated time point, the results showed that Snail protein levels, as well as inhibitory GSK-3β phosphorylation, were significantly reduced, and E-cadherin expression was markedly increased (Fig. 4B). Furthermore, reduced cell invasion was observed following GF109203X treatment (Fig. 4C). These data indicate that the increased E-cadherin expression in LoVo-P cells transfected with the exogenous miR-203a-3p mimic is due to Snail protein degradation, which is mediated by decreased PKC activity and subsequently increased GSK-3β activity.

### Expression of miR-203a-3p and E-cadherin in vivo

**Fig. 5:**
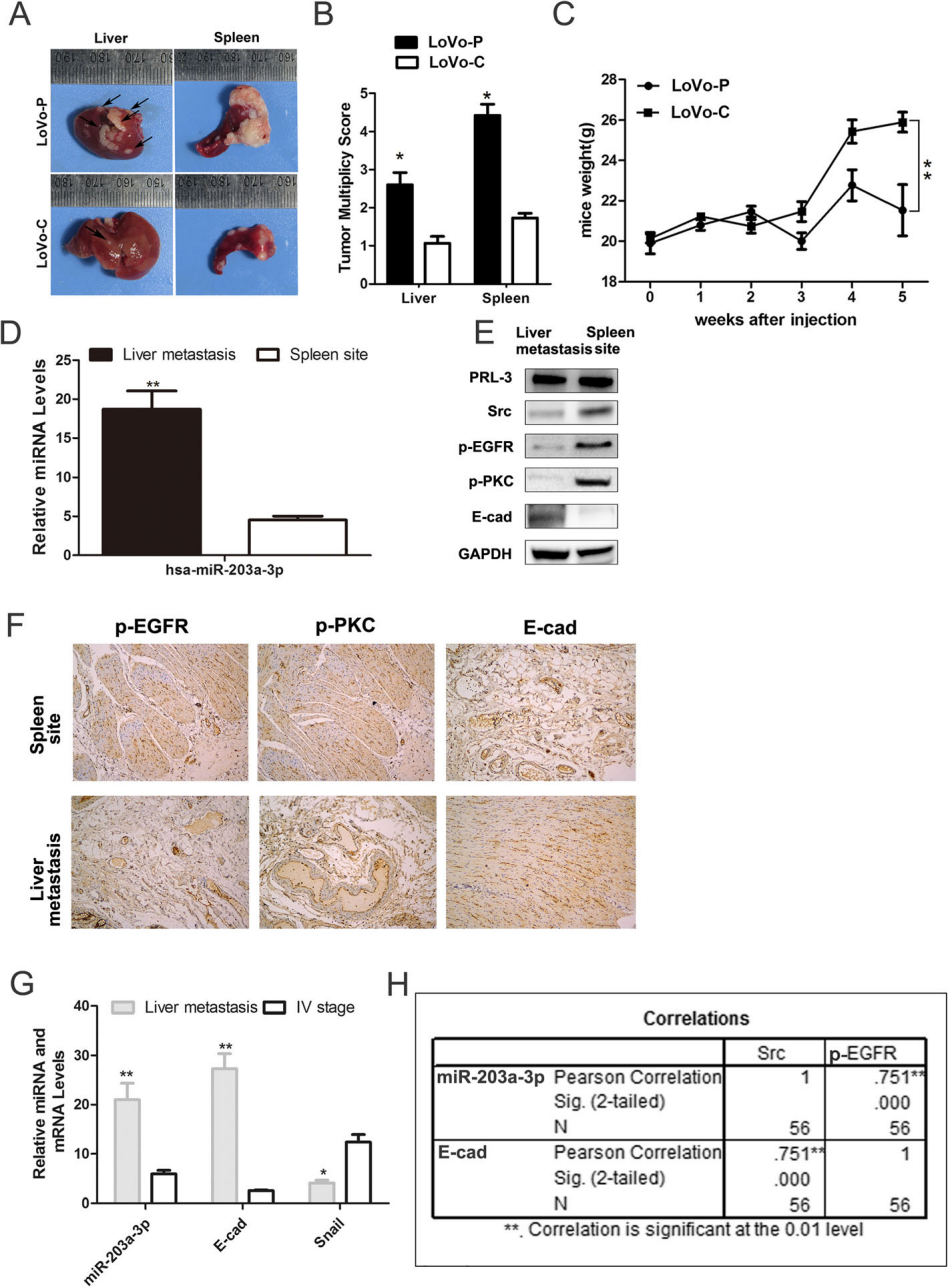
Expression of miR-203a-3p and E-cadherin in vivo. BALB/c-nu/nu female mice, 6- to 8-weeks-of-age, were divided randomly into 2 groups, separately injected LoVo-P / LoVo-C in spleens. (A) Image of tumor in spleen and liver. (B-C) Liver metastasis score and weight of mice. qPCR (D) and western blot assays (E) examining the expression of miR-203a-3p, PRL-3, Src, p-EGFR, p-PKC and E-cadherin in the spleen site and liver metastases. (F) IHC examining the p-EGFR, p-PKC and E-cadherin immunoreactivity in the spleen site and liver metastases. (G) Expression of miR-203a-3p/E-cadherin (E-cad)/Snail in the primary site and liver metastases. (H) Correlation between miR-203a-3p/E-cadherin (E-cad) and Src/p-EGFR expression

In vivo animal model experiments were performed to examine the effects of PRL-3 on tumor metastasis. LoVo-P cells were administered to athymic nude mice via intrasplenic injection. More liver metastasis nodules were observed in the LoVo-P cell-injected group than in the LoVo-C cell-injected groups (Fig. 5A-C). In addition, E-cadherin and miR-203a-3p expression was detected in the metastasis sites (Fig. 5D, E). Moreover, the results showed that the expression levels of p-EGFR and p-PKC were decreased in the metastasis sites (Fig. 5E). In 30 human CRC liver metastases samples, we performed IHC and qPCR analyses of miR-203a-3p, E-cadherin and Snail expression. The results showed that miR-203a-3p expression was similar to E-cadherin expression, but Snail was rarely expressed, which revealed the relationship between miR-203a-3p and E-cadherin/Snail (Fig. 5F-H). These results demonstrate the correlation between miR-203a-3p and E-cadherin/Snail in liver metastases.

## Discussion

Metastasis is the primary cause of death in patients with cancer, which is a multistep process including the detachment of cancer cells from the primary site and their transport into the blood vessels and colonization to distal organs; this process allows tumor cells to disseminate from their primary site and establish secondary tumors at secondary sites. Research has demonstrated that EMT is the main mechanism of this process [29]. Once tumor cells colonize distal organs, mesenchymal-to-epithelial transition (MET) may occur. However, the mechanism of successive EMT and MET switches remains unclear, and proposed mechanisms remain under discussion [30]. In this study, our research found that hepatocytes induced E-cadherin re-expression in PRL-3-overexpressing CRC cells, and this re-expression of E-cadherin is considered a marker of MET. Furthermore, miR-203a-3p from the exosomes of hepatocytes was found to inhibit Src expression and EGFR activation in CRC cells and to promote E-cadherin re-expression. In summary, our research determined that hepatocyte-derived miR-203a-3p induced MET in PRL-3-overexpressing CRC cells.

PRL-3 belongs to the protein-tyrosine phosphatase family, which plays a unique role in signal transduction. Researchers have noted that PRL-3 expression is higher in CRC metastases than in primary colorectal tumors and normal colon tissues, indicating that PRL-3 promotes the liver metastasis of CRC [30]. Numerous studies have revealed that the PI3K/AKT signaling pathway, integrin signaling pathway and Rho signaling pathway are the molecular mechanisms underlying cancer metastasis induced by PRL-3 [31]. Furthermore, PRL-3 could recruit endothelial cells to promote angiogenesis [32]. Our previous research demonstrated that PRL-3 promotes CRC cell EMT, which is a major step in CRC cell liver metastasis. However, less is understood about how metastases are formed.

Exosomes are important for intracellular communication. Increased knowledge of the function of exosomes in cancer progression implies that the exosomal process is an orchestrated process. For example, research using the chorioallantoic membrane of chick embryos demonstrated that tumor cell xenografts within the membrane required exosome release for directional movement [33]. Exosomes have a vast array of contents, which are composed of microRNAs, mRNA transcription factors and proteins and are highly variable and depend on cell origin [34]. MicroRNAs are small, non-coding RNAs that upon entering the recipient cell, bind to the target mRNA sequence and inhibit translation. Research has found that miRNAs can be used as diagnostic and prognostic biomarkers [35]. The shuttling of miRNA molecules that are either tumor-supportive or tumor-suppressive is important in cancer. In our research, we found that miR-203a-3p secreted by hepatocyte-derived exosomes specifically binds Src and inhibits Src expression and EGFR activation. MiR-203a-3p has been reported playing an important role in a variety of cancers. In hepatocellular carcinoma, it promotes HCC cell proliferation and metastasis [36]. In Barrett’s esophagus cells, up-regulating miR-203a-3p can inhibit cell proliferation [37]. Interestingly, it was found miR-203a-3p promoted proliferation, colony formation, apoptosis, invasion and migration by suppressing the expression of PDE4D in CRC [38], we hypothesis mir-203a-3p may has different effects on CRC in different organs. The Src family of kinases comprises nine structural non-receptor tyrosine kinases. The activity of Src is increased in most solid tumors and some hematologic malignancies and is positively correlated with progressive stages of cancer [39]. Research has found that the tyrosine phosphorylation of β catenin and Tiam 1 by Src suppresses the association of β catenin with E-cadherin and disrupts the integrity of cell-to-cell junctions [40]. In addition, Src expression and activation in cancer cells always occurs after alterations to one or more of its activators, such as EGFR [41]. Interestingly, a study also revealed that PRL-3 could induce EGFR hyperactivation [15]. EGFR is a transmembrane receptor tyrosine kinase that is overexpressed in 49–82% of CRC. EGFR overexpression could induce tumor cell EMT and then promote the progression and metastases of colorectal carcinoma [42]. To date, study has suggested that the inhibition of autocrine EGFR signaling increases E-cadherin expression and promotes EMT in human prostate carcinoma upon co-culture with hepatocytes [43]. In our research, Src was inhibited by miR-203a-3p, and EGFR activation and E-cadherin expression were inhibited; these findings are consistent with former results and indicate that hepatocytes promote high levels of PRL-3 expression in CRC cell MET through miR-203a-3p binding to Src and inhibiting EGFR activation.

To further detect the mechanism for MET in co-cultured CRC, we also detected Snail expression. Snail is a zinc-finger transcription factor that has been proven to directly bind to the E-boxes of the E-cadherin promoter and repress its expression. The data show that LoVo cell Snail expression levels were decreased, indicating that the accumulation of Snail was responsible for the repression of E-cadherin. It is well known that Snail is a substrate of GSK-3β. GSK-3β could phosphorylate Snail and promote its degradation through the ubiquitin-proteasome pathway. Previous research has also proven that PKC kinase could phosphorylate GSK-3β. In this study, we found that PRL-3 could induce the activity of PKC. When the PKC inhibitor GF 109203X was used, the phosphorylation of GSK-3β induced by PKC was completely inhibited, leading to the degradation of Snail. The data indicated that the down-regulation of the PKC/GSK-3β/Snail pathway was responsible for MET in CRC cells co-cultured with hepatocytes.

In summary, our study indicates that miR-203a-3p derived from hepatocyte exosomes plays an important role in promoting MET by inhibiting Src expression. The down-regulation of Src resulted in the inhibition of EGFR activation and the downstream signaling pathways and thus reduced the expression of E-cadherin, these results reveal the mechanism of liver metastases formation by CRC cells, which provides comprehensive insight into CRC liver metastasis.

## Data Availability

The data used and/or analyzed during the current study are available from the corresponding author on reasonable request.

## Abbreviations

CRC: Colorectal Cancer cells
ZEB1: Zinc finger E-box Binding homeobox 1
ZEB2: Zinc finger E-box Binding homeobox 2
DMEM: Dulbecoo’s Modified Eagle Medium
PBS: Phosphate Buffer Saline
PKC: Protein Kinase C
VEGF: Vascular Endothelial Growth Factor
EGFR: Epidermal Growth Factor Receptor
RIPA: Radio Immunoprecipitation Assay
PMSF: Phenylmethanesulfonyl Fluoride
GSK-3β: Glycogen synthase kinase-3β
